# Effects of the source gap on transmission efficiency of a quadrupole mass spectrometer

**DOI:** 10.1002/rcm.8094

**Published:** 2018-04-14

**Authors:** Mariya J. Antony Joseph, David G. McIntosh, J. Raymond Gibson, Stephen Taylor

**Affiliations:** ^1^ Mass Spectrometry and Instrumentation Group, Department of Electrical Engineering and Electronics University of Liverpool Brownlow Hill Liverpool L69 3GJ UK

## Abstract

**Rationale:**

Recent trends towards miniature and portable quadrupole mass spectrometry (QMS) entail challenges in instrumental sensitivity, which is influenced by 3D fringe field effects on ion transmission in the Quadrupole Mass Filter (QMF). The relationship of these effects with the gap from the ion source to the QMF entrance (source gap) is significant and little explored. We examine transmission characteristics experimentally and use the results to test the predictive accuracy of a recently developed 3D QMF simulation model. The model is then applied to directly investigate optimal transmission m/z ranges across multiple source gaps.

**Methods:**

A portable single filter quadrupole mass spectrometer is used to analyse transmission characteristics across a range of common gases. We use an experimental approach originally proposed by Ehlert, enhanced with a novel method for absolute calibration of the transmission curve. Custom QMF simulation software employs the boundary element method (BEM) to compute accurate 3D electric fields. This is used to study the effects of the source gap on transmission efficiency.

**Results:**

Experimental findings confirm a centrally peaked transmission curve; simulations correctly predict the optimal transmission location (in m/z) and percentage, and extend the experimental trend. We compare several methods for determining fringe field length, demonstrating how the size of the physical source gap influences both the length and the intensity of the fringe field at the QMF entrance. A complex relationship with ion transmission is revealed in which different source gaps promote optimal transmission at differing m/z ranges.

**Conclusions:**

The presented results map the relationship between the source gap and transmission efficiency for the given instrument, using a simulation method transferrable to other setups. This is of importance to miniature and portable quadrupole mass spectrometers design for specific applications, for the first time enabling the source gap to be tailored for optimal transmission in the desired mass range.

## 1 INTRODUCTION

The Quadrupole Mass Filter (QMF) is widely used in analytical instruments, with applications from biomedical science to environmental monitoring.^1^, ^2^ There is an increasing trend towards the development of small and miniature portable quadrupole mass spectrometers for point of use applications.^3^, ^4^, ^5^, ^6^, ^7^, ^8^, ^9^, ^10^ Sensitivity is an important and longstanding constraint, especially for miniature instruments.^11^ To retain performance with small or micro footprint instruments, highly accurate modelling of QMF behaviour for various design geometries is advantageous since it is capable of streamlining experimental prototype testing.

Following previous practice, the phrase 'ion transmission' is used to refer to the peak maxima of mass scans. The 'transmission efficiency' of a QMF refers to the proportion of target ions (i.e. ions having *m/z* values corresponding to the peak maximum of the mass scan) which, having left the source, are successfully transmitted through the QMF.^12^ Analysis of QMF behaviour assuming an ideal quadrupole field (that is, a field resulting from infinite length electrodes, referred to as '2D') takes electric field components in all three co‐ordinate directions (*E*_*x*_, *E*_*y*_, *E*_*z*_) to be independent of *z* position, with *E*_*z*_ = 0.^13^ Such ideal analysis predicts that as long as stable ions experience sufficient radiofrequency (RF) cycles in the QMF, transmission efficiency does not depend upon *m/z*.^14^

**Figure 1:**
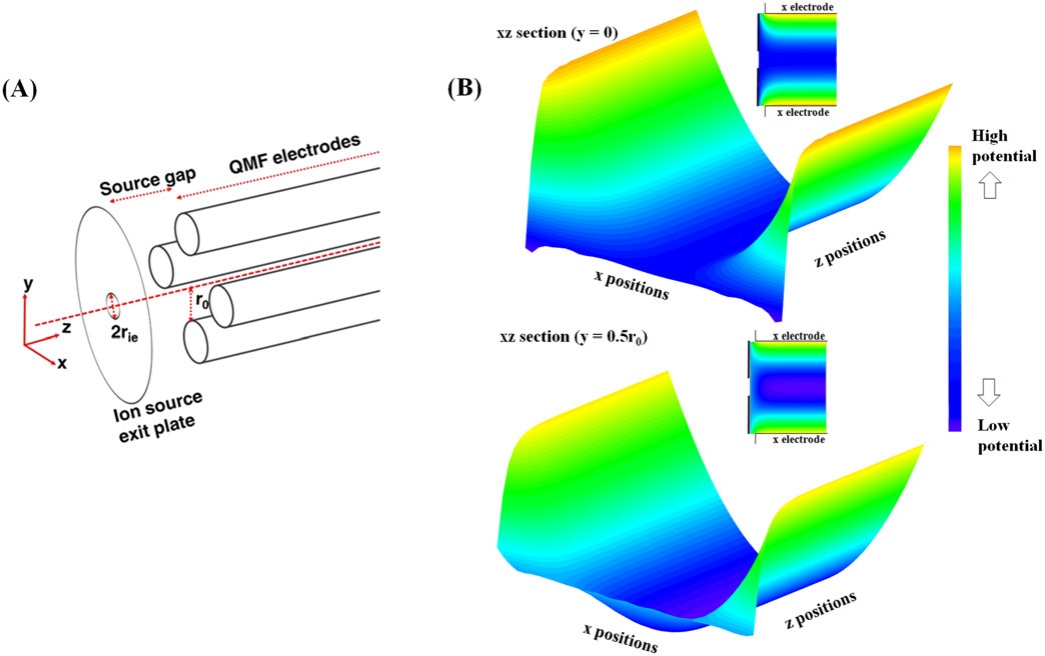
(A) Schematic diagram of the Quadrupole Mass Filter (QMF) with four electrodes of constant cross section, whose axes are parallel with, and equally spaced around, the *z* axis, touching an inscribed circle of radius *r*_0_. The gap between the source end‐plate and the QMF electrodes is referred to as the 'source gap’. (B) *xz* equipotential plots (3D and 2D representations) for a fringe field region with a small source gap (0.25*r*_0_), produced using potential values from the custom simulation model. Representations are at two elevations in *y*, and potential contours are shown up to an axial displacement of 3*r*_0_ from the ion source exit plate [Color figure can be viewed at http://wileyonlinelibrary.com]

In practice, fields are 3D, having non‐zero *E*_*z*_, and all field components subject to change with *z* position because of finite length electrodes and earthed end plates coupling the ion source and detector to the QMF (Figure 1A). The resultant fringing electric fields at the entrance and exit of the QMF (see Figure 1B) have a mass‐dependent influence on transmission efficiency for a given ion energy and frequency. Due to the fringe field influence on position, velocity and relative field phase, there can be effective defocusing, resulting in loss of signal,^15^ or focusing,^16^ with a corresponding effect on instrument sensitivity for different *m/z*.

QMF fringe fields are complex because 3D field distribution causes coupling of ion motion in the *x*, *y* and *z* directions. Comparison of the two equipotential plots of Figure 1B illustrates a single example of coupled ion motion: the initial direction of the force exerted by the *z* component of the field on positive ions (towards or away from the ion source) at a given phase angle is dependent on proximity to the *y* electrode.

### 1.1 Fringe fields and transmission efficiency

**Figure 2:**
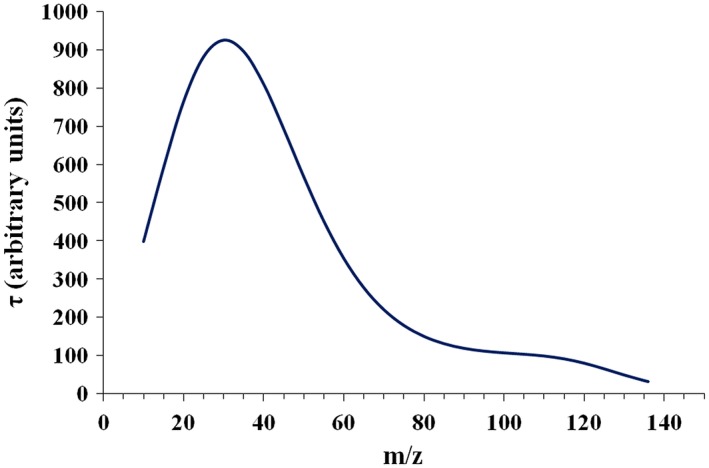
QMF transmission curve as a function of *m/z* generated from Ehlert's results^18^ [Color figure can be viewed at http://wileyonlinelibrary.com]

Few experimental results have been published that treat the effect of fringe fields on ion transmission.^12^, ^17^ Early theoretical and experimental work found that QMF ion transmission efficiency will fall for heavier ions which spend a longer period exposed to certain defocusing forces in the *y* direction, occurring because the typical operating point lies outside the *y*‐stability limit for large portions of the fringe field region.^12^, ^17^ Following this, Ehlert employed an experimental approach based on isotope ratio measurements to plot ion transmission efficiency as a function of *m/z*.^18^ We know of no other comparable attempt. The resulting curve (see Figure 2) showed the expected fall in transmission at higher *m/z*,^12^, ^17^ but transmission unexpectedly peaked near *m/z* 30 and fell at lower *m/z*; this fall was believed at the time to be an artefact of the RF detection circuitry of the instrument. Following this, however, an approximate linear model of the fringe field (in which *E*_*x*_ and *E*_*y*_ ramp linearly from zero to full quadrupole fields on attaining a prescribed displacement from the starting position of the ions, typically *r*_0_)^16^, ^19^, ^20^ predicted a transmission fall‐off at lower *m/z* using phase space dynamics. It was concluded that QMF mass discrimination effects may be predicted as a function of the number of RF cycles (optimal between 1 and 3 RF cycles) spent by ions of different *m/z* in the fringe field around the entrance to the QMF.

Hennequin and Inglebert investigated this model experimentally.^21^ Study of their 50% acceptance ellipses reveals a range of agreement with the predictions of linear approximation model from good to poor, depending on the number of cycles spent by ions in the fringe field (*n*_*f*_), and whether acceptance in *x* or *y* was considered. In these experiments, *n*_*f*_ was altered by means of ion *m/z* and energy. The implementation of the linear approximation model assumed a geometric fringe field length (*l*_*f*_) equal to *r*_0_.^16^ Independent determination of *l*_*f*_ was not attempted; the authors observed that this length should be set on the basis of experimental results.

Subsequent workers employed a Liebmann iterative relaxation technique to generate an approximation of the fringing field having an exponential form for variation of *E*_*x*_, *E*_*y*_ and *E*_*z*_ with *z* position.^22^ A cubic version was developed for phase‐space analysis and tested against the experimental findings of Hennequin and Inglebert.^23^ In the implementation of the cubic model, *l*_*f*_ was set at 1.5*r*_0_, and *n*_*f*_ was varied by means of the ions' axial velocities, with a fixed source gap of 0.125*r*_0_. Overall improvement in the linear approximation was marginal, despite arguable improvements in *y*‐acceptance for some axial velocities.

An advantage of the exponential model is the avoidance of discontinuous transition to zero *E*_*z*_ at the prescribed distance from the source plate. However, the model implies perpetuation of ideal quadrupole field symmetry (between *E*_*x*_ and *E*_*y*_) in the fringe field, corresponding to a lack of compliance with Laplace's Equation. Further, an inherent difficulty of phase‐space analysis is that one must neglect the coupling of ion motion in all three dimensions caused by non‐zero *E*_*z*_ to avoid complex six‐dimensional phase‐space calculations.^16^ The significant remaining discrepancies with experimental data were therefore tentatively ascribed to perturbations in axial velocity (*v*_*z*_).

### 1.2 Motivation

Most previous studies have been principally concerned with testing the soundness of theoretical models rather than investigating the transmission effects of varying either the fringe field length or the source gap. In these cases, the fringe field length in terms of number of RF cycles was altered by means of the ion's initial axial velocity (either directly, or using ion mass or energy). This is not equivalent to altering either the geometric fringe field length or the source gap, since (a) altering axial velocity affects not just the number of cycles in the fringe field but also the number of cycles that ions experience in the QMF itself, and thus the resolution; (b) the fringe field length (in number of RF cycles) changes proportionately with the axial velocity of ions, but disproportionately with the source gap; and (c) the fringe field position with respect to the QMF remains the same (other than being scaled in number of RF cycles) when the axial velocity is altered, while the fringe field distribution alters such that it penetrates further into the QMF, when the physical source gap is reduced.

The source gap is a parameter that may easily be altered at the design stage, and can be used to influence transmission efficiency without recourse to ion energy alterations that may be unavailable or counterproductive. A simulation model has recently been developed which accurately and efficiently reproduces 3D field effects (including fluctuations in axial velocity) and can simulate variations in both the source gap and the fringe field length.^24^, ^25^ Remaining disparities between existing models of QMF behaviour and experimental observation, and the lack of direct investigation of source gap effects, suggest the potential benefits of an investigation with such a high‐fidelity model.

Generation of an experimental transmission curve would be advantageous in light of the single previous example,^18^, ^21^ both to compare QMF transmission characteristics obtained with a different set of instrumental parameters (particularly the transmission reduction at lower *m/z* originally questioned by Ehlert^18^) and to assess the accuracy of the simulation model. We also explore a method of fixing the absolute transmission efficiency (not attempted by Ehlert). Once the accuracy of the simulations is established, these can be extended to predict the effects at a wider range of *m/z* and to investigate variation of the physical source gap. Any such attempt should explicitly define how the fringe field length is determined (we have recently proposed such a method^25^), and clearly distinguish between the mass selection implications of the fringe field length, and those of the source gap length. We accurately characterise for the first time the relationship of QMF transmission characteristics to these connected but distinct parameters, and use individual ion trajectories to investigate the more complex and realistic picture which emerges.

## 2 EXPERIMENTAL

### 2.1 Method

Measurements were made using a commercial single filter portable QMS instrument (supplied by Q‐Technologies, Liverpool, UK) incorporating an electron ionisation source with a single focus, and a dual detection apparatus (Faraday plate and electron multiplier). The instrument has a mass range from *m/z* 1 to approximately *m/z* 200. The geometrical dimensions are: *r*_0_ = 2.753 mm; *r* (radius of circular electrodes) = 1.148*r*_0_ (3.16 mm); *r*_*ie*_ = 0.334*r*_0_ (0.92 mm); source gap = detector gap = 0.726 *r*_0_ (~2 mm); detector end‐plate aperture radius = 0.581*r*_0_ (1.6 mm); electrode length (*l*) ≈ 45*r*_0_ (125.4 mm), and frequency of rf supply (*f*) = 2 MHz. The system is housed in a stainless steel vacuum chamber with a residual (background) gas total pressure of ~4 × 10^−6^ Pa. The ion gauge was calibrated for N_2_ throughout experiments. This use of a single pressure gauge calibration for all gases is in keeping with Ehlert's original experiments,^18^ although not strictly optimal for quantitative comparison of signals. Research grade neon, argon, krypton and xenon gas (BOC Ltd, Manchester, UK) were individually supplied to the vacuum chamber, raising the ion gauge reading to ~7 × 10^−3^ Pa in each case, with an ion injection energy of 5 eV.

Ehlert's method^18^ was followed to produce a transmission efficiency curve for this instrument (enhanced by a method to determine absolute transmission efficiency). The method compares measured and known isotopic abundances to establish transmission efficiency ratios $\tau_{s_{1}}/\tau_{s_{2}}\;$between pairs of isotopes of several elements, where S_1_ and S_2_ refer to isotopic species 1 and 2 of the same element S. The approach assumes that the ion source behaviour will be the same for both isotopes. Equation (1) then allows comparison of the ratio in detected ion current ( $I_{s_{2}}/I_{s_{2}})\;$with the known isotopic ratio (*A*_s1_/*A*_*s*2_) to determine the transmission efficiency ratio of the QMF for each pair of isotopes:

(1) $$\frac{\tau_{S1}}{\tau_{S2}}=\frac{I_{S1}A_{S2}G_{S2}}{I_{S2}A_{S1}G_{S1}}$$

where *G*_S1_ and *G*_S2_ are the gains of the detector used for the respective species. Provided that the same detector gain is used for two isotopes of the same element, then in Faraday mode G_S1_/*G*_S2_ = 1, while in multiplier mode, it has been shown^26^ that:

(2) $$\frac{G_{S1}}{G_{S2}}=\sqrt{\frac{{\left(m/z\right)}_{S2}}{{\left(m/z\right)}_{S1}}}$$

It is assumed that the transmission curve can be approximated by an equation whose logarithm takes the form of Equation (3), if a suitable order can be chosen:

(3) $$\mathrm{lg}\tau =C_{0}+C_{1}\left(\frac{m}{z}\right)+C_{2}{\left(\frac{m}{z}\right)}^{2}+\ldots$$

With the exception of *C*_0_, all polynomial coefficients (*C*_1_, *C*_2_, etc.) are found through least squares curve fitting of Equation (4) (which follows from Equation (3)) for all isotopic pairs:

(4) $$\mathrm{lg}\frac{\tau_{S1}}{\tau_{S2}}=C_{1}\left\{{\left(\frac{m}{z}\right)}_{S1}\right.-\left.{\left(\frac{m}{z}\right)}_{S2}\right\}+C_{2}\left\{{{\left(\frac{m}{z}\right)}^{2}}_{S1}\right.-\left.{{\left(\frac{m}{z}\right)}^{2}}_{S2}\right\}+\ldots$$

The choice of *C*_0_ then sets the scale of the transmission curve generated by substituting *C*_1_ to *C*_7_ back into Equation (3) and raising it to an exponent of 10. Ehlert^18^ chose *C*_0_ arbitrarily to give *τ* = 100 at *m/z* = 0. In our case, RF‐only transmission data were compared to determine *τ* empirically as a percentage of the total ion current entering the QMF (Appendix A).

### 2.2 Simulation method

Gibson et al have demonstrated that the Boundary Element Method (BEM) is both an accurate and a computationally efficient method of calculating QMF 3D electric field values.^27^ In this work, we have used a simulation model which utilises the BEM to compute accurate field values in the fringe field regions and throughout the QMF. A full description of the method, including examples of 2D and 3D computed spectra, can be found in the literature.^24^, ^25^ Using this approach, it becomes possible to plot the variation of ion transmission through the QMF as a function of customised operating parameters and dimensions. A significant advantage of the model is the ability to compute individual trajectories of a large number of randomly injected ions (up to ~10^7^) that combine to generate statistically significant, detailed mass spectral predictions.

The model treats the motion of ions from the moment of emergence from the source plate central aperture, as they traverse the source gap, QMF and detector gap. Ions reaching the central aperture of the detector end‐plate are considered successfully detected. The QMF dimensions (*r*_0_, *r*, *r*_*ie*_, source and detector gaps, detector end‐plate aperture radius, and *l*) and frequency of RF waveform were chosen to be the same as those of the experimental setup. The model was calibrated to the experimental QMS resolution by setting the *U*/*V* ratio (an expression for the percentage of the optimal ratio of DC to RF voltage, which is 0.16784:1) to a constant value of 99.5% across all measurements. Together, these measures allow prediction of the effects of fringe fields on ion transmission for a QMF as similar as possible to the experimental setup.

## 3 RESULTS AND DISCUSSION

### 3.1 Ehlert's transmission curve

Figure 2 is a recalculation of Ehlert's smooth transmission curve as a function of *m/z* using the original ion current and isotope transmission ratio data (well described by Ehlert^18^) and a similar least‐squares algorithm (7^th^ order polynomial fit with standard error in log *τ* of 0.0017 and an R^2^ of 0.9995.) The value of *C*_0_ was set to 2 in accordance with the value chosen arbitrarily by Ehlert, which if extended creates an intercept of *τ* = 100 for his experimental curve. This is not to be confused with a percentage value; it is rather a (hypothetical) prediction of relative transmission efficiency at zero *m/z*, created by extrapolation of the fitted curve. Such an intercept value, relative or otherwise, should be treated as a feature of the curve fit rather than a necessary prediction of the experimental data. A similar argument applies to the points calculated from Equation (5) to generate the curve. For these reasons, we have refrained from Ehlert's original practice of extending the curve below *m/z* 10 (the lowest measured *m/z*) or showing the calculated points.

### 3.2 Experimental transmission curve

**Figure 3:**
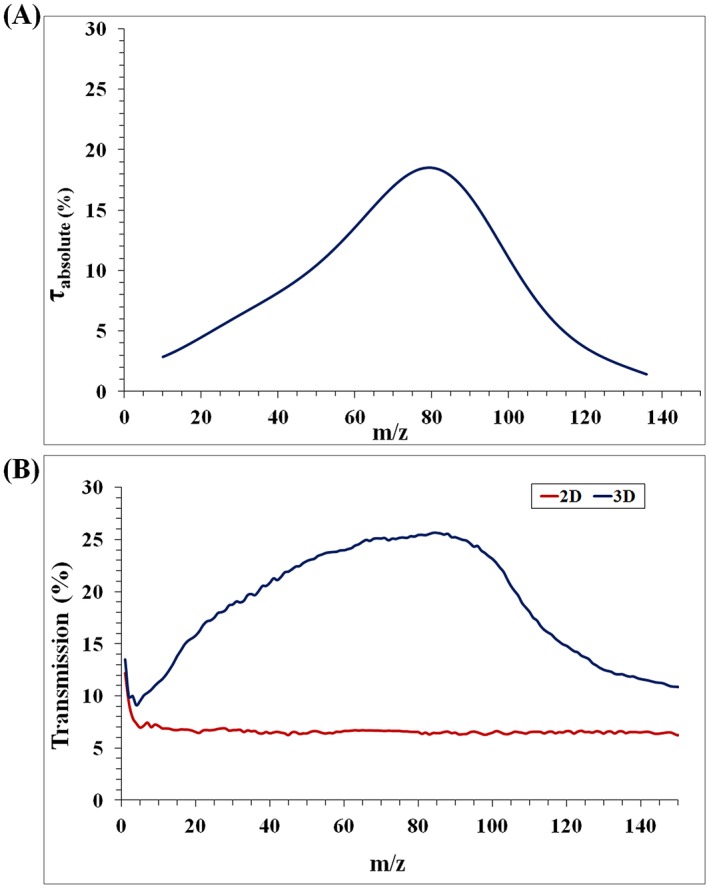
QMF transmission curves as a function of *m/z*: (A) absolute transmission curve generated from isotope measurements taken on our instrument (detailed in Table 1) and (B) ion transmission curves using 2D and 3D simulation methods [Color figure can be viewed at http://wileyonlinelibrary.com]

For the new transmission curve (Figure 3A), neon, argon, krypton and xenon were introduced into the quadrupole mass spectrometer inlet separately from pressurised cylinders under uniform operational pressure, source voltage and other instrumental operating conditions including *U*/*V* ratio. Measurements were taken at mass steps of 0.01 *m/*z units. A minimum time interval of 3 to 5 h was usually allowed to elapse between measurements taken from different gases; in these cases, the instrument was allowed to reach its base pressure before introducing the subsequent gas. Immediately before introducing each gas, residual gases in the chamber (where apparently present in significant quantity) at the *m/z* of the target analytes were measured for possible subtraction from the target signal. However, even when present, the residual gas component was found to be negligible in its effect.

Table 1 provides the ion transmission ratios (*τ*_S1_/*τ*_S2_) of isotopic pairs of different species calculated from known abundance ratios (*A*_S2_/*A*_S1_)^28^, ^29^ using Equation (1). The instrument was operated mostly in Faraday mode, making the detector gain factor equal to unity;^18^ in other cases, Equation (2) was employed to correct for mass‐selective multiplier effects.

**Table 1:** New experimental transmission ratios obtained for different isotopic pairs

Species (S)	(*I*_S1_/*I*_S2_)*(*G*_S2_/*G*_S1_)	*A*_S2_/*A*_S1_	*τ*_S1_/*τ*_S2_
^20^Ne^2+^, ^22^Ne^2+^	9.337	0.102	0.952
^20^Ne^+^, ^22^Ne^+^	9.102	0.102	0.928
^36^Ar^+^, ^40^Ar^+^	0.003	298.573	0.896
^80^Kr^2+^, ^86^Kr^2+^	0.125	7.559	0.945
^84^Kr^2+^, ^86^Kr^2+^	3.212	0.303	0.973
^129^Xe^2+^, ^134^Xe^2+^	2.405	0.395	0.951
^134^Xe^2+^, ^136^Xe^2+^	1.153	0.849	0.979
^78^Kr^+^, ^80^Kr^+^	0.151	6.439	0.972
^80^Kr^+^, ^82^Kr^+^	0.201	5.071	1.019
^84^Kr^+^, ^86^Kr^+^	3.421	0.303	1.037
^129^Xe^+^, ^132^Xe^+^	1.171	1.019	1.193
^134^Xe^+^, ^136^Xe^+^	1.37	0.849	1.163

By procuring coefficients *C*_1_ to *C*_7_ as described in section 2, an equation for the relative transmission *τ* was obtained as a function of *m/z*:

(5) $$\mathrm{lg}\tau =C_{0}-8.580\times 10^{-4}\left(\frac{m}{z}\right)+2.157\times 10^{-3}{\left(\frac{m}{z}\right)}^{2}-9.961\times 10^{-5}{\left(\frac{m}{z}\right)}^{3}+2.134\times 10^{-6}{\left(\frac{m}{z}\right)}^{4}-2.331\times 10^{-8}{\left(\frac{m}{z}\right)}^{5}+1.242\times 10^{-10}{\left(\frac{m}{z}\right)}^{6}-2.565\times 10^{-13}{\left(\frac{m}{z}\right)}^{7}$$

Figure 3A shows the absolute transmission efficiency curve produced from Equation (5) following calculation of *C*_0_. (Appendix A; note that neither the shape of the curve nor the *m/z* location of maximum transmission efficiency is influenced by *C*_0_.)

As with Ehlert's results,^18^ a seventh‐order polynomial approximation was used for the curve fit of log *τ*, with, in the case of the new data, a standard error of 0.007 and an R^2^ value of 0.9836. The order of polynomial used affects the shape and accuracy of the curve fit. It is entirely possible that a lower order polynomial could give a poorer fit at the data points but result in a better interpolation between the data points and hence a more accurate relative transmission curve; this is particularly relevant to *m/z* regions with few experimental data points. We investigated lower order polynomials to order 3 (standard error 0.012; R^2^ = 0.922.) Above order 3 there was no effect on the *m/z* position of peak transmission, while for order 3, the optimal transmission position was shifted slightly to *m/z* 85. The effect on peak shape was more noticeable: reducing the polynomial order produced a broader, flatter transmission peak, with (below order 6) elimination of the slight hump on the low *m/z* side. Overall, the minor impact appeared to support the robustness of the generated curve.

Ehlert's method^18^ is also potentially susceptible to small measurement errors. Careful subtraction of baseline measurements and occasional deconvolution of overlapping peaks are important while calculating the ion current ratios. Ion current ratios are affected by pressure fluctuations and voltage drift, and so an average of typically 10 peak transmission measurements was obtained over a short period at uniform pressure for each element. Analytical error for ion current ratios across a given element ranged between 1 and 2.5% relative standard deviation (RSD); calculations involving small peaks were prone to higher error than those with larger peaks. To study the impact on the transmission curve, a random error of up to ±2.5% was artificially introduced into the recorded ion current ratios. This resulted in shifting of the peak of the transmission curve by ~ ±5 *m/z* units and small alterations in the shape of the curve.

Figure 3A bears out the general trend shown by Ehlert's experiments taken on a different quadrupole mass spectrometer, with transmission initially rising to a peak as *m/z* increases, before falling away, and reaffirms the fall in transmission efficiency observed by Ehlert^18^ at lower *m/z*. The transmission peak occurs at a different *m/z* position (*m/z* ~80), as does the overall optimal transmission range; this is attributable to the different instrument parameters such as source gap, electrode length, *r*_0_, frequency of RF waveform, ion energy and resolution settings,^24^, ^25^ and points to the limits of generalising experimental results from a single instrument.

### 3.3 Absolute transmission efficiency

To convert the relative transmission curve predicted by Equation (5) into absolute transmission efficiency (*τ*_*absolute*_) as shown in Figure 3A, a reliable estimate for the scaling constant *C*_0_ was required. To achieve this, several local estimates of *C*_0_ were calculated, each for a given isotope/charge state (referred to as a component) of a different element, by substituting a value (*X*_*i*_) representing the absolute ion transmission efficiency for component *i*, into Equation (5) in place of *τ*. *X*_*i*_ was obtained by ascertaining the ratio of the measured ion current *I*_2*i*_ (obtained in mass scanning mode with non‐zero DC component) with the measured ion current for zero DC (RF‐only mode). The latter may be taken to represent the input current, *I*_1*i*_, of component *i* at the source aperture. That is:

(6) $$X_{i}=\frac{I_{2i}}{I_{1i}}$$

Obtaining the ratio *I*_2*i*_/*I*_1*i*_ is not a trivial exercise. Attempting this directly by choosing the local peak in the RF‐only spectrum representing *I*_1*i*_ was found to be a complex process subject to human error and the precise calibration of the instrument's mass scale. Reliability is further reduced because the determination of *I*_1*i*_ for a single isotope introduces significant additional analytical error. The proportions of doubly ionised peaks increased the complexities. A more feasible approach was therefore formulated (fully described in Appendix A) using the reasonable assumption that the maximum RF‐only ion current measured during introduction of a single element represents the total ion current at the source (*I*_1_) of that element. Using relative transmissions already available from Equation (5), we applied correcting factors to the measured ion currents for all *N* components. These were then summed and divided by the peak RF‐only value to obtain an estimate of *X*_*i*_ for the component under consideration (for example, *X*_1_):

(7) $$X_{1}=\frac{I_{21}+\sum_{i=2}^{N}I_{2i}\lambda_{1i}}{I_{1}}$$

Substitution of *X*_*i*_ into Equation (5) for *τ*, the calculated *C*_0_ is identical regardless of which component of the given element is used, but will, in practice, vary by element due to analytical error. Comparison of the calculated *C*_0_ values showed strong agreement between all elements except xenon, which was an outlier predicting higher transmission efficiency. This was taken to indicate the likelihood of misalignment of sample pressure between the mass spectra and RF‐only spectra for xenon. Such a misalignment, while not necessarily affecting the transmission ratios obtained for the components of xenon, could artificially inflate the absolute transmission calculation. It was therefore decided to use the average of the values obtained for neon, argon and krypton, *C*_0_ =  − 1.673, for absolute calibration of the transmission curve as shown in Figure 3A.

The effect of the order of polynomial curve fit on the absolute transmission curve was investigated. For orders 6 to 3, *C*_0_ varies between −1.848 and −1.588; the effect on the peak value of the absolute transmission efficiency is <1%.

### 3.4 Simulated transmission curve

To test the predictions of our simulation model, particularly in terms of the maximum transmission efficiency and its location on the *m/z* scale, we simulated ion transmission as a function of *m/z* for the experimental instrument, with and without fringe field effects. This also allows investigation of ion transmission at very low *m/z*, which is difficult to measure experimentally assuming typical ion energies and, as discussed in the previous section, is not possible to predict confidently from experimental data at higher *m/z* using a curve‐fitting equation. To minimise statistical variations while still allowing time‐efficient computation, 20,000 ion trajectories were simulated at each mass step of 0.02 *m/z* units. The results described here are for a precisely centered source plate aperture, emitting a uniformly distributed beam of ions having identical energy (5 eV), random distribution of initial RF phase angle and travelling parallel to the system axis. This allowed us to determine the effects of varying some of these parameters, the results of which are available in Figure S1 (supporting information).

Figure 3B shows the simulated ion transmission as a function of *m/z* with and without fringe field effects. For the results without fringe field effects (ideal quadrupole 2D fields, red line), the ion transmission reduces sharply with the initial *m/z* increase, and then remains constant. The results including 3D fringe field effects show a similar transmission curve to that obtained experimentally by Ehlert's method^18^ in Figure 3A, including a very similar peak transmission position (albeit slightly flatter and broader) and a similar *m/z* range of overall optimal transmission. The absolute percentage of transmission is moderately higher than that calculated experimentally; this could be symptomatic of imperfect setting of simulation parameters (e.g. spread in initial ion energy and angle) or experimental imperfections (e.g. isotopic fractionation effects or a slightly off‐axis source plate aperture).^30^, ^31^ Similar factors could be responsible for the slight differences in curve shape. Furthermore, as discussed in section 2, Ehlert's approach^18^ was followed in this work, which used a single gauge calibration for all gases. Preliminary investigations suggest that the differences in shape would be reduced by calibrating the gauge separately to each gas. Overall, the level of agreement in terms of order of magnitude suggests merit in the RF‐only comparison method of fixing absolute transmission and supports the fundamental accuracy of the simulation method. Comparison between the 2D and 3D simulated curves confirms that the transmission peak is purely due to the effect of 3D fringe fields.

While the broad trends of previous models are borne out,^12^, ^16^ with a pattern of transmission reductions at both lower and higher *m/z*, the BEM also predicts clear fluctuations (non‐smooth) in ion transmission which are not a result of statistical variations as discussed in our previous work.^24^, ^25^ Furthermore, the simulation model indicates a sharp rise in transmission below *m/z* 4 which was difficult to investigate experimentally. This is to be expected in the case of ions travelling parallel to the system axis since, as *m/z* tends to zero, their initial velocity must tend to infinity, guaranteeing their arrival at the detector. The trend is reduced, but still apparent, for a small initial half angle between the ion beam and the system axis, as seen in Figure S1 (supporting information). For the specific quadrupole mass spectrometer dimensions and RF deployed in this study, experimental and simulation evidence agree that optimal ion transmission occurs when the QMF is operated at a range from about *m/z* 60 to 90.

### 3.5 Fringe field length (*l*_*f*_)

Many earlier works have assumed for convenience a fringe field length (*l*_*f*_) of fixed distance equal to *r*_0_.^16^, ^20^ While the fringe field length is impossible to determine since it never completely disappears even in the region between the QMF electrodes, there will be a distance into this region where its effects become negligible. We have previously described an approach to determining an effective *l*_*f*_ by ascertaining a boundary at which both $\frac{\partial E_{x}}{\partial x}$ and $\frac{\partial E_{y}}{\partial y}$ fall to 99.9% of their maximal, constant value further inside the QMF region (near‐perfectly quadrupole fields). The practical usefulness of this method was supported by favourable comparison with a method in which ion trajectories began at a variable distance from the source plate and looked for the threshold distance at which fringe field effects became negligible.^25^

The field gradient change occurs at significantly different lengths along different axial lines; one approach to determining a single value for the field error is to measure the gradient of a least‐squares fit of the field (e.g. *E*_*x*_) across a range of values of *x* and *y* within 0.5*r*_0_ of the QMF axis. Calculation of *l*_*f*_ in the current work required, instead, a 0.1% reduction from ideal field gradients to have been individually reached or surpassed across the vast majority of the region within 0.5*r*_0_ of the QMF axis.

The results of this method for calculating *l*_*f*_ were compared with two other approaches. In the first comparison, the *z* boundary at which the potential falls by 0.1% across many axial lines within 0.5*r*_0_ of the QMF axis was calculated (Figure S2, supporting information). In the second, since the absolute value of *E*_*z*_ must initially increase (from zero) with the corresponding reductions in *E*_*x*_ and *E*_*y*_ when travelling towards the source plate, the *z* boundary at which |*E*_*z*_| reduces below a threshold value along many axial lines was used. The variation of |*E*_*z*_| is not monotonic in the *z* direction, and the values of |*E*_*z*_| attained vary according to the source gap. This threshold value was therefore chosen as 0.1% of the average of maximum *E*_*z*_ across the vast majority of the region within 0.5*r*_0_ of the system axis and across all source gaps (±1.25 × 10^−4^ *V*/*m*).

Table 2 summarises the results of the three approaches. The fringe field lengths determined agree well with one another. *l*_*f*_ is identical for the source gaps 0.25*r*_0_ and 0.125*r*_0_, in agreement with the findings of previous studies, although taking a slightly higher value.^23^

**Table 2:** Fringe field length data based on (A) 0.1% reduction from near perfect quadrupole fields of $\frac{\partial E_{x}}{\partial x}$ and $\frac{\partial E_{y}}{\partial y}$, (B) 99.9% reduction from the average maximum *E*_*z*_ magnitude, and (C) 0.1% fall in potentials from their values in the near perfect quadrupole region

Source gap (*r*_0_ units)	$\frac{\partial E_{x}}{\mathrm{\partial x}}$ method (*r*_0_ units)	$\frac{\partial E_{y}}{\mathrm{\partial y}}$ method (*r*_0_ units)	***E***_***z***_ method (*r*_0_ units)	Potential method (*r*_0_ units)
0.125	1.7	1.7	1.8	1.8
0.25	1.7	1.7	1.8	1.8
0.5	1.8	1.8	1.9	1.9
0.75	1.9	1.9	2.1	2
1	2.2	2.2	2.3	2.2
1.5	2.6	2.6	2.8	2.7
2	3.2	3.2	3.3	3.2

### 3.6 Source gap effect and fringe field length

**Figure 4:**
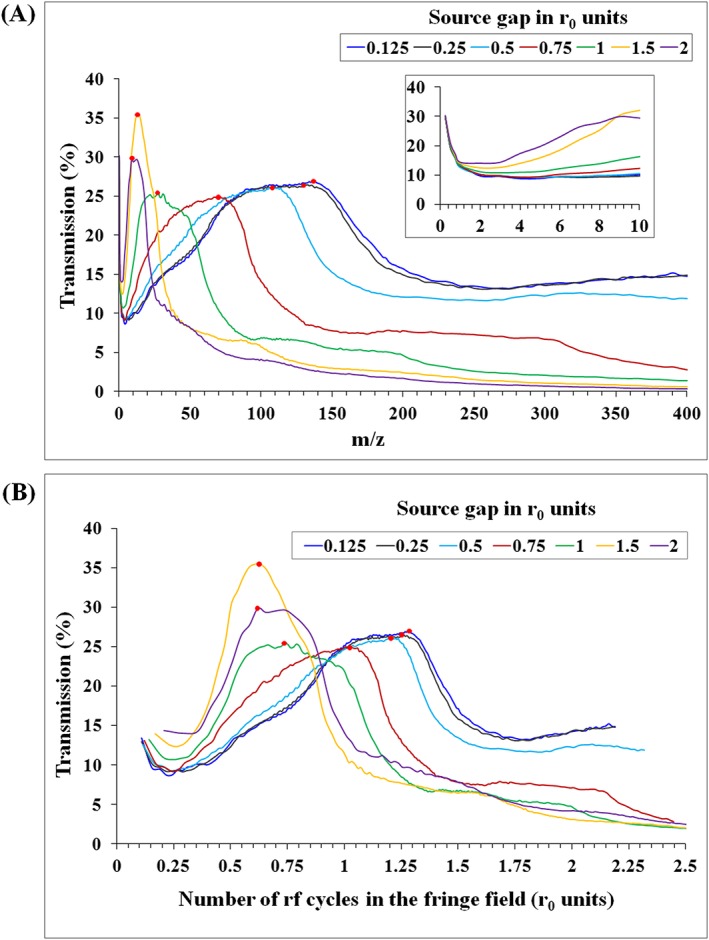
The effect of source gap length on ion transmission: (A) as a function of *m/z* and (B) as a function of the number of RF cycles the ion spends in the fringe field. (Fringe field length calculated using a gradient change percentage threshold for *E*_*x*_ and *E*_*y*_)

Figure 4 shows the transmission results of simulating six source gap lengths from 0.125*r*_0_ to 2*r*_0_ (QMF dimensions and operating parameters are otherwise identical to those of Figure 3B). Varying the source gap affects the intensity of the fringe field around the QMF entrance, its length, and its degree of penetration into the QMF. These factors significantly alter the influence of the fringe field on ion transmission. Beneficial transmission effects change when the source gap length is altered, such that the ion's arrival conditions (QMF entry positions, angle and velocity) are important in addition to the general relationship between the number of RF cycles spent in the fringe field and ion transmission.

Figure 4A shows the source gap influence as a function of *m/z*. The peak position of the transmission curve (signified by a red dot) is dependent on the length of the source gap when other parameters are held constant. The peak shift in the transmission curve for source gaps below 0.25*r*_0_ is negligible; this agrees with earlier predictions of minimal difference in fringe field length for source gaps less than 0.25*r*_0_.^23^ Optimal transmission shifts to lower *m*/*z* as the source gap increases; the optimal position shows greatest mobility for source gaps between 0.5*r*_0_ and *r*_0_. Increasing the source gap beyond 1.5*r*_0_ significantly compromises the transmission but with little change to the optimal *m*/*z* range. It is also noticeable that the spread of *m*/*z* experiencing optimal transmission reduces as the source gap length is increased. There is little appreciable difference in the transmission peak height (i.e. optimal transmission efficiency) for source gaps ≤*r*_0_. The optimum value increases markedly for a source gaps of 1.5*r*_0_ and reduces again for a 2*r*_0_ gap.

High transmission with a long source gap will be reduced if ions are introduced with an angle spread (Figure S3, supporting information), but the trend and peak position remain the same. The effects predicted in Figure 4A are also similar for a QMF with hyperbolic electrodes (Figure S4, supporting information). In general, the height of the transmission curve tends to reduce slightly for source gaps increasing from 0.25*r*_0_ to *r*_0_. The peak of the transmission curve will also shift by changing ion energies but the trend in transmission curve height and shape differs from the source gap change.

Figure 4B shows the ion transmission as a function of the approximate number of RF cycles spent by ions in the fringe field, *N*_*f*_ (*r*_0_ units). *N*_*f*_ may be calculated by: $N_{f}=l_{f}f\sqrt{\frac{m}{2\epsilon_{z}e}}$,^19^ where *l*_*f*_ is the length of the fringe field, *f* is the frequency of the RF voltage, *m* is the mass of the ion, *ϵ*_*z*_ is the accelerating voltage of the ion source in the *z* direction and *e* is electron charge. This method of calculating *N*_*f*_ technically relies on the assumption of constant axial velocity in the fringe field, as practised by the majority of previous workers in this field. However, a cursory comparison with the actual number of cycles predicted by accurate simulations across multiple source gaps, *m/z* and RF phase angles suggested that the equation serves reasonably well, i.e. that fluctuations in axial velocity do not have a major effect on fringe field dwell times.

The results presented in Figure 4B show that peak transmission for all source gaps occurs in fringe field dwell times between ~0.6*r*_0_ and 1.3*r*_0_ RF cycles, in accordance with the previous analysis.^16^, ^19^, ^25^ While this approximate general relationship between *N*_*f*_ and transmission efficiency is clear, the specific range of *N*_*f*_ for optimal transmission is dependent on the individual source gap. Usually increasing the source gap promotes optimal transmission for ions experiencing smaller number of RF cycles in the fringe field. The effect of the source gap on the optimal transmission position with respect to *N*_*f*_ (highlighted by red dots) is most significant between gaps of 0.25*r*_0_ and 1.5*r*_0_.

**Figure 5:**
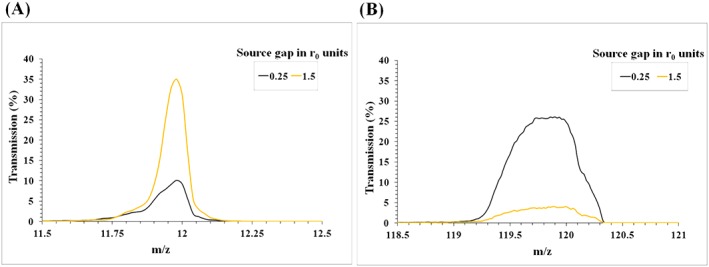
The effect of source gap length on ion transmission for individual mass peaks of high and low *m/z* [Color figure can be viewed at http://wileyonlinelibrary.com]

Smaller source gaps are clearly beneficial for optimal transmission at higher *m*/*z*, and vice versa. To illustrate a potential application of these findings, the simulated spectra in Figure 5 show that a smaller source gap of ~0.25*r*_0_ is beneficial for transmission efficiency of stable ions with *m/z* 120 (for the given QMF settings), while, for a low‐mass ion such as *m/z* 12, a larger source gap of ~1.5*r*_0_ is beneficial. It is clear that the improvement in transmission is not at the expense of resolution in either case. Optimising the source gap is a critical component of QMS design for mass‐specific applications;^32^, ^33^ in a modern twist on the conclusions of Hennequin and Inglebert,^21^ accurate simulation of QMF behaviour for the specific instrument under consideration is a key to realising the most appropriate source gap design.

## 4 CONCLUSIONS

The predictions of 3D simulations have been tested using Ehlert's method of measuring the variation of transmission with *m/z* in a quadrupole mass spectrometer. Relative transmission values generated in this way were converted into absolute values by an RF‐only comparison approach devised to exploit the Ehlert method in the case of double ionisation. Good agreement has been found between simulations and experimental results, although further experimental testing on an instrument with a moveable source gap would strengthen this conclusion, and will be the subject of future work.

Having confirmed the validity of the simulations, we investigated the effect of the source gap on the variation of transmission with *m/z*. Accurate 3D simulations enabled precise mapping of the relationship between the source gap and the region of optimal transmission. Smaller source gaps promote optimal transmission at higher *m/z* without the need for increased ion energies. It is possible to predict in detail the optimum source gap length for transmission at a given range of *m*/*z* when designing and manufacturing an instrument for specific application. These conclusions are particularly significant for portable and miniature instruments, where sensitivity is a key concern.

## Supporting information


**Figure S1:** The effect of different ion entry conditions on transmission as a function of *m/z* values for the same QMF settings and operating conditions as in Figure 3(B) with source and exit gaps ~ 2 mm
**Figure S2:** Potential plots (2D representations) in yz (at x=0.5r_0_) for QMF with three different source gaps (0.25r_0_, 0.75r_0_ and 1.5r_0_) are shown to an axial displacement of 3*r*_0_ from the ion source exit plate using the custom simulation model for a typical fringe field region.
**Figure S3:** The effect of source gap length on ion transmission as a function of *m/z* for the same QMF settings and operating conditions as in Figure 4(A); except, ions exiting the source exit plate with angular spread of +/‐ 5^o^
**Figure S4:** The effect of source gap length on ion transmission as a function of *m/z* for the same QMF with hyperbolic electrodes. Dimensions and other operating conditions including parallel ion entry conditions are same as in Figure 4(A)

## APPENDIX A

### A.1

Let us assume that the maximum RF‐only ion current measured, during introduction of a single element, represents the total ion current at the source, *I*_1_, of this element. *I*_1_ comprises the currents of *N* isotopic species including its doubly ionised variants (referred to as components and denoted by the subscript *i*); that is:

(8) $$I_{1}=\sum_{i=1}^{N}I_{1i}$$

These several components of *I*_1_ occur for different *m/z* and thus are subject to the mass‐selective relative transmission effects approximated by Equation (5). If *I*_2*i*_ refers to the detected ion current for component *i* in mass‐scanning mode, our goal is to calculate the absolute transmission efficiency *X*_*i*_ = *I*_2*i*_/*I*_1*i*_ for a given component.

Equation (5) yields *τ*_*i*_, the relative transmission at the *m/z* of component *i*:

(9) $$\tau_{i}=cX_{i}=cI_{2i}/I_{1i}$$

where $c=10^{-C_{0}}$ is an unknown scale factor. Performing the same computation for any other component *j* of the same element, we can calculate *λ*_*ji*_, the ratio of the transmission for component *j* to that of component *i*:

(10) $$\lambda_{\mathrm{ji}}=\frac{\tau_{j}}{\tau_{i}}=\frac{\left(I_{2j}/I_{1j}\right)}{\left(I_{2i}/I_{1i}\right)\;}$$

Rearranging Equation (9), substituting the result for *I*_1*i*_ into Equation (8) , multiplying by *τ*_1_/*c* and comparing the result with Equations (10) and (9) yields *X*_1_:

(11) $$I_{1}\frac{\tau_{1}}{c}=\frac{\tau_{1}}{c}\sum_{i=1}^{N}\frac{{\mathrm{cI}}_{2i}}{\tau_{i}}=I_{21}+\sum_{i=2}^{N}I_{2i}\left(\frac{\tau_{1}}{\tau_{i}}\right)=I_{21}+\sum_{i=2}^{N}I_{2i}\lambda_{1i}\;\Rightarrow \;X_{1}=\frac{I_{21}+\sum_{i=2}^{N}I_{2i}\lambda_{1i}}{I_{1}}$$

*X*_1_ can be substituted in Equation (5) for *τ* to obtain a component‐specific value for *C*_0_:

(12) $$C_{0}=\mathrm{lg}\left(X_{1}/\tau_{1}\right)$$

## References

[1] Dawson PH , Hedman JW , Whetten NR . A miniature mass spectrometer. Anal Chem. 1970;42(12):103A‐108A.

[2] Maher S , Jjunju FP , Taylor S . Colloquium: 100 years of mass spectrometry: Perspectives and future trends. Rev Modern Phys. 2015;87(1):113‐135.

[3] Malcolm A , Wright S , Syms RR , et al. Miniature mass spectrometer systems based on a microengineered quadrupole filter. Anal Chem. 2010;82(5):1751‐1758. 20108919 10.1021/ac902349k

[4] Wright S , Malcolm A , Wright C , et al. A microelectromechanical systems‐enabled, miniature triple quadrupole mass spectrometer. Anal Chem. 2015;87(6):3115‐3122. 25708099 10.1021/acs.analchem.5b00311

[5] Ding JP , Zhou KH , Tang LX , et al. Miniature quadrupole mass spectrometer array and its applications in manned spaceflight. Space Med Med Eng (Beijing). 2004;17(1):74‐78. 15005118

[6] Chen CH , Chen TC , Zhou X , et al. Design of portable mass spectrometers with handheld probes: aspects of the sampling and miniature pumping systems. J Am Soc Mass Spectrom. 2015;26(2):240‐247. 25404157 10.1007/s13361-014-1026-5 PMC4323736

[7] Emary WB , Isern‐Flecha I , Wood KV , Ridley TY , Cooks RG . Desorption ionization/tandem mass spectrometry with a caesium ion source and a triple quadrupole mass spectrometer. Talanta. 1986;33(12):1001‐1007. 18964244 10.1016/0039-9140(86)80241-7

[8] Jjunju FP , Maher S , Li A , et al. Hand‐held portable desorption atmospheric pressure chemical ionization ion source for in situ analysis of nitroaromatic explosives. Anal Chem. 2015;87(19):10047‐10055. 26329926 10.1021/acs.analchem.5b02684

[9] Taylor S , Tunstall JJ , Leck JH , et al. Performance improvements for a miniature quadrupole with a micromachined mass filter. Vacuum. 1999;53(1‐2):203‐206.

[10] Taylor S , Gibson J , Srigengan B . Miniature mass spectrometry: implications for monitoring of gas discharges. Sensor Rev. 2003;23(2):150‐154.

[11] Boumsellek S , Ferran RJ . Trade‐offs in miniature quadrupole designs. J Am Soc Mass Spectrom. 2001;12(6):633‐640. 11401154 10.1016/S1044-0305(01)00248-3

[12] Brubaker WM. Auxiliary electrodes for quadrupole mass filters. US Patent 3129327. 14 Apr. 1964.

[13] Miller PE , Denton MB . The quadrupole mass filter – Basic operating concepts. J Chem Ed. 1986;63(7):617‐622.

[14] Gibson JR , Taylor S . Prediction of quadrupole mass filter performance for hyperbolic and circular cross section electrodes. Rapid Commun Mass Spectrom. 2000;14(18):1669‐1673. 10962488 10.1002/1097-0231(20000930)14:18<1669::AID-RCM80>3.0.CO;2-#

[15] Douglas DJ . Linear quadrupoles in mass spectrometry. Mass Spectrom Rev. 2009;28(6):937‐960. 19492304 10.1002/mas.20249

[16] Dawson P . The acceptance of the quadrupole mass filter. Int J Mass Spectrom Ion Phys. 1975;17(4):423‐445.

[17] Brubaker WM . An improved quadrupole mass analyzer. Adv Mass Spectrom. 1968;4293‐4299.

[18] Ehlert TC . Determination of transmission characteristics in mass filters. J Phys E: Sci Instrum. 1970;3(3):237‐239.

[19] Dawson PH . Quadrupole Mass Spectrometry and Its Applications. Amsterdam: Elsevier; 1976.

[20] Dawson P . Ion optical properties of quadrupole mass filters. Adv Electronics Electron Phys. 1980;53:153‐208.

[21] Hennequin JF , Inglebert RL . Experimental‐study on acceptance of a quadrupole mass filter. Int J Mass Spectrom Ion Processes. 1978;26(2):131‐135.

[22] Hunter KL , McIntosh BJ . An improved model of the fringing fields of a quadrupole mass filter. Int J Mass Spectrom Ion Processes. 1989;87(2):157‐164.

[23] McIntosh BJ , Hunter KL . Influence of realistic fringing fields on the acceptance of a quadrupole mass filter. Int J Mass Spectrom Ion Processes. 1989;87(2):165‐179.

[24] Gibson JR , Evans KG , Syed SU , Maher S , Taylor S . A method of computing accurate 3D fields of a quadrupole mass filter and their use for prediction of filter behavior. J Am Soc Mass Spectrom. 2012;23(9):1593‐1601. 22777712 10.1007/s13361-012-0426-7

[25] Gibson JR , Evans KG , Taylor S . Predicted behaviour of QMF systems with and without prefilters using accurate 3D fields. Int J Mass Spectrom. 2017;422197‐422207.

[26] Ploch W , Walcher W . Correction in mass‐spectrometric abundance measurements in consequence of the mass dependence of the electron‐release by means of isotope ions. Rev Sci Instrum. 1951;22(12):1028‐1028.

[27] Gibson JR , Evans KG , Taylor S . Modelling mass analyzer performance with fields determined using the boundary element method. J Mass Spectrom. 2010;45(4):364‐371. 20198605 10.1002/jms.1720

[28] Böhlke J , De Laeter J , De Bievre P , et al. Isotopic compositions of the elements, 2001. J Phys Chem Ref Data Monogr. 2005;34(1):57‐67.

[29] Berglund M , Wieser ME . Isotopic compositions of the elements 2009 (IUPAC Technical Report). Pure Appl Chem. 2011;83(2):397‐410.

[30] Muntean F . Transmission study for rf‐only quadrupoles by computer simulation. Int J Mass Spectrom Ion Processes. 1995;151(2‐3):197‐206.

[31] Taylor S , Gibson JR . Prediction of the effects of imperfect construction of a QMS filter. J Mass Spectrom. 2008;43(5):609‐616. 18076125 10.1002/jms.1356

[32] Ellefson RE , Moddeman WE , Dylla HF . Hydrogen isotope analysis by quadrupole mass‐spectrometry. J Vacuum Sci Technol. 1981;18(3):1062‐1067.

[33] Schneider B , Kuiper K , Postma O , Wijbrans J . Ar‐40/Ar‐39 geochronology using a quadrupole mass spectrometer. Quater Geochronol. 2009;4(6):508‐516.

