# Supplementary material for: Effects of the source gap on transmission efficiency of a quadrupole mass spectrometer
Source: Rapid Commun Mass Spectrom. 2018 Apr 14;32(9):677–85. doi: 10.1002/rcm.8094 (PMC5947150; doi:10.1002/rcm.8094)

# Effects of the source gap on transmission efficiency of a quadrupole mass spectrometer

Mariya J. Antony Joseph, David McIntosh, Ray Gibson and Stephen Taylor^*^

Mass Spectrometry and Instrumentation Group, Department of Electrical Engineering and Electronics, University of Liverpool, UK

*** Address reprint requests to:**

Professor Stephen Taylor;

Mass Spectrometry and Instrumentation Group

Department of Electrical Engineering and Electronics,

University of Liverpool,

Brownlow Hill, Liverpool,

L69 3GJ,

UK.

Email: [S.Taylor@liv.ac.uk](mailto:S.Taylor@liv.ac.uk)

## Supporting document : supplementary figures

**Figure S1:** The effect of different ion entry conditions on transmission as a function of *m/z* values for the same QMF settings and operating conditions as in Figure 3(B) with source and exit gaps ~ 2mm

**
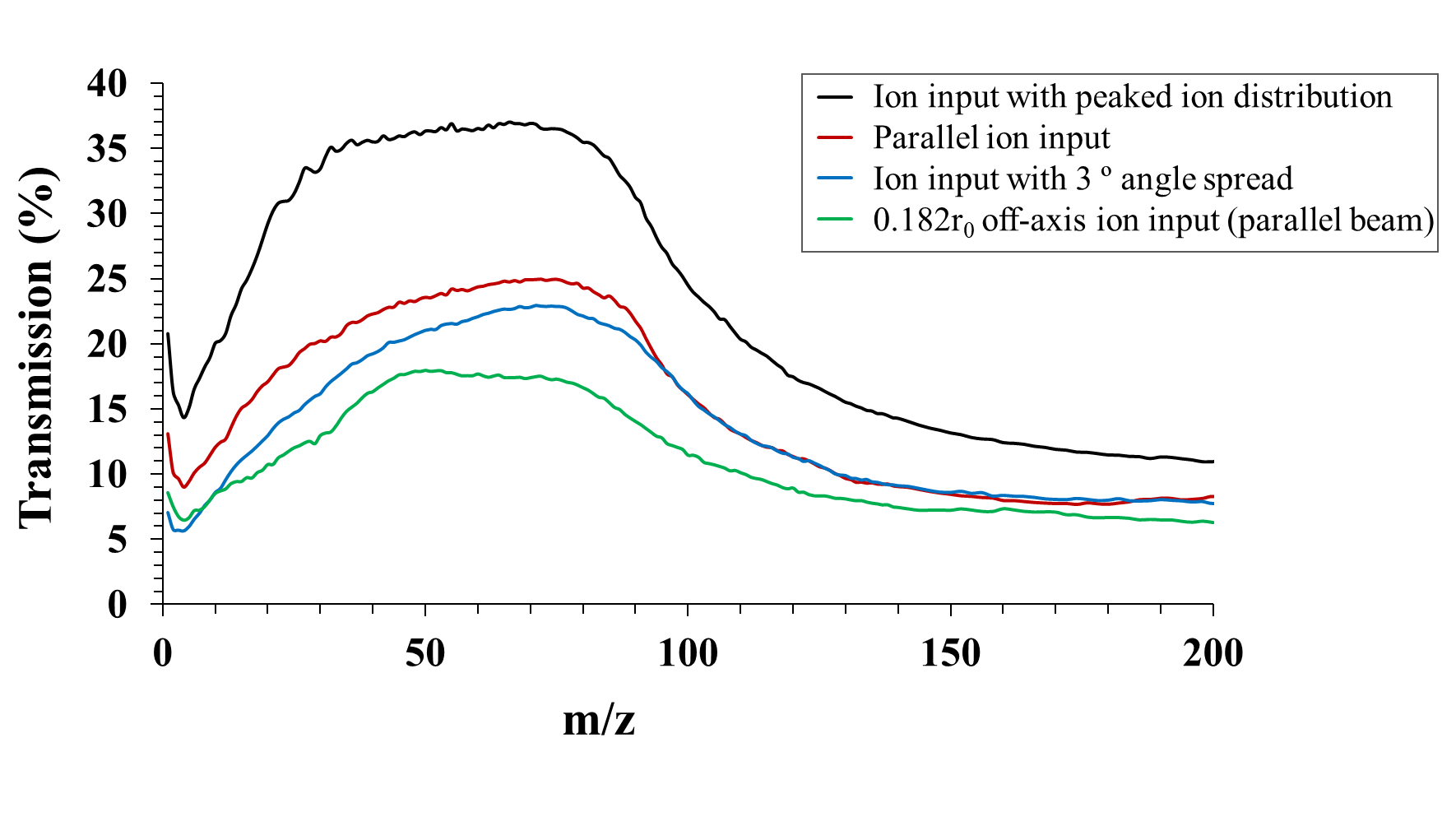
**

**Figure S2:** Potential plots (2D representations) in yz (at x=0.5r_0_) for QMF with three different source gaps (0.25r_0_, 0.75r_0_ and 1.5r_0_) are shown to an axial displacement of $3r_{0}$ from the ion source exit plate using the custom simulation model for a typical fringe field region.


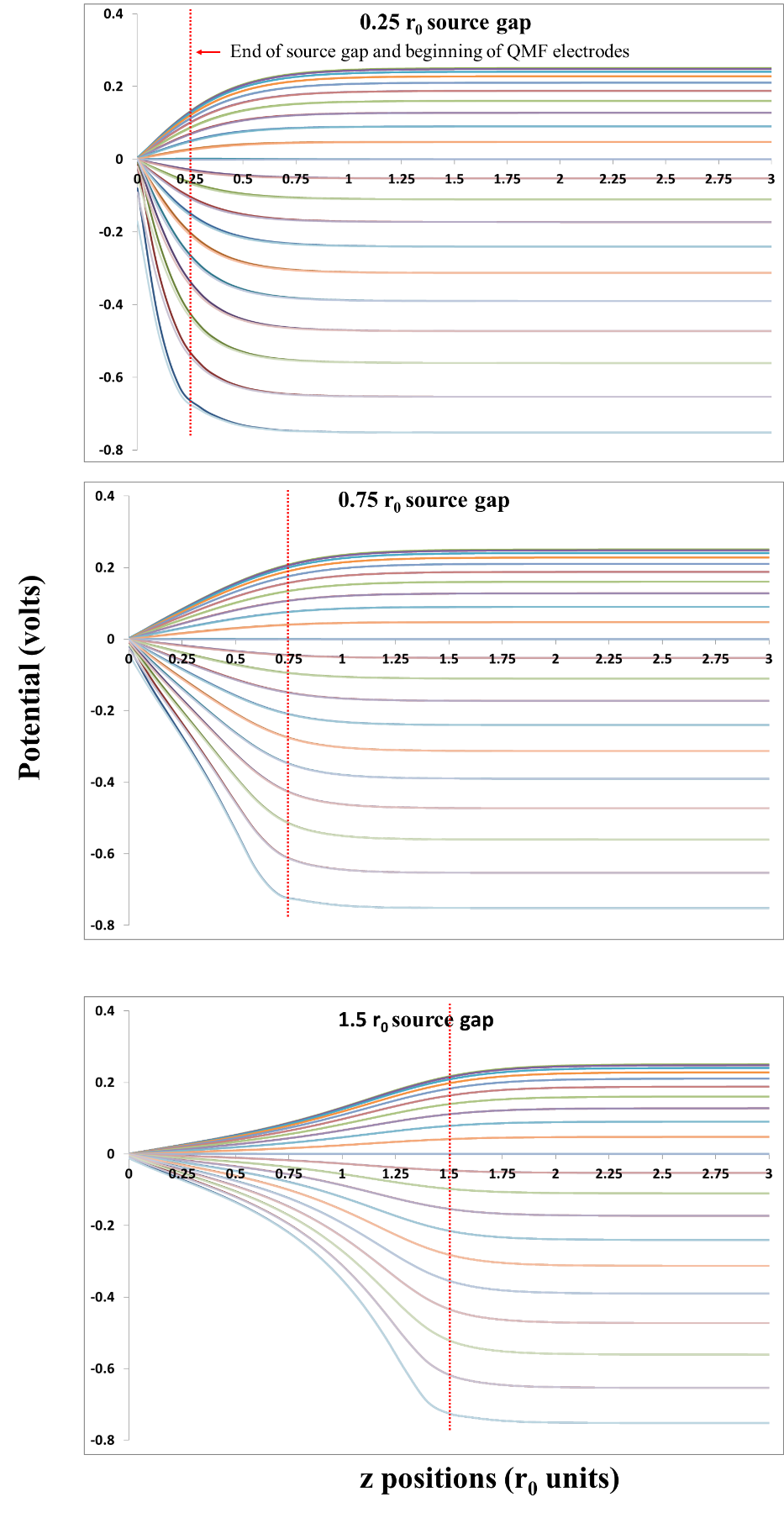


**Figure S3:** The effect of source gap length on ion transmission as a function of *m/z* for the same QMF settings and operating conditions as in Figure 4(A); except, ions exiting the source exit plate with angular spread of +/- 5^o^


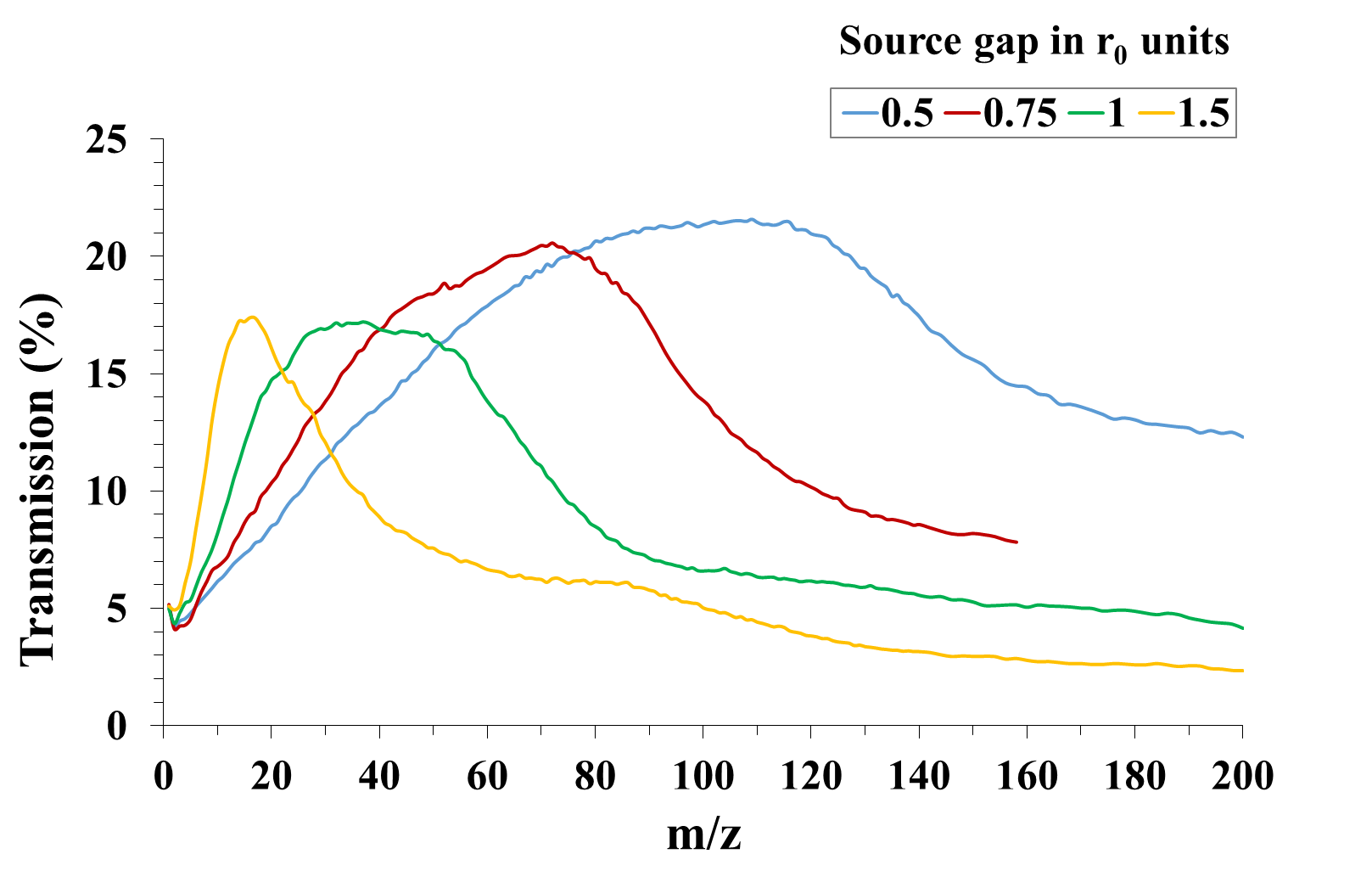


**Figure S4:** The effect of source gap length on ion transmission as a function of *m/z* for the same QMF with hyperbolic electrodes. Dimensions and other operating conditions including parallel ion entry conditions are same as in Figure 4(A)


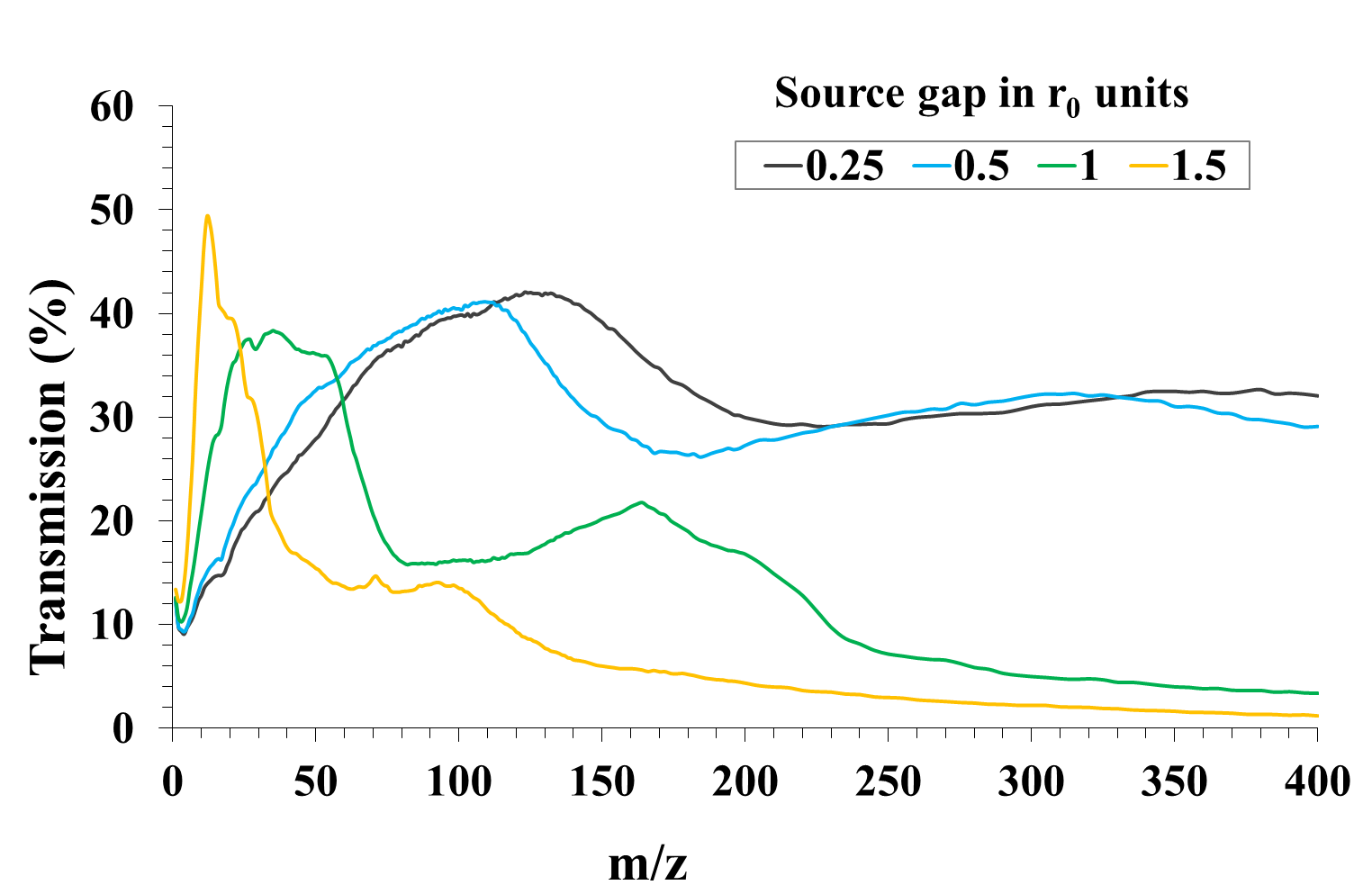

Supplement: Supplementary file 1 — Figure S1: The effect of different ion entry conditions on transmission as a function of m/z values for the same QMF settings and operating conditions as in Figure 3(B) with source and exit gaps ~ 2 mm Figure S2: Potential plots (2D representations) in yz (at x=0.5r0) for QMF with three different source gaps (0.25r0, 0.75r0 and 1.5r0) are shown to an axial displacement of 3r0 from the ion source exit plate using the custom simulation model for a typical fringe field region. Figure S3: The effect of source gap length on ion transmission as a function of m/z for the same QMF settings and operating conditions as in Figure 4(A); except, ions exiting the source exit plate with angular spread of +/‐ 5o Figure S4: The effect of source gap length on ion transmission as a function of m/z for the same QMF with hyperbolic electrodes. Dimensions and other operating conditions including parallel ion entry conditions are same as in Figure 4(A) [file RCM-32-677-s001.docx]
